# Thrombus Structural Composition in Cardiovascular Disease

**DOI:** 10.1161/ATVBAHA.120.315754

**Published:** 2021-07-15

**Authors:** Ghadir Alkarithi, Cédric Duval, Yu Shi, Fraser L. Macrae, Robert A.S. Ariëns

**Affiliations:** 1 Discovery and Translational Science Department, Leeds Institute of Cardiovascular and Metabolic Medicine, University of Leeds, United Kingdom (G.A., C.D., Y.S., F.L.M., R.A.S.A.).; 2 Department of Medical Laboratory Technology, Faculty of Applied Medical Sciences, King Abdulaziz University, Jeddah, Saudi Arabia (G.A.).

**Keywords:** blood vessels, cardiovascular diseases, myocardial infarction, thrombectomy, thrombosis

## Abstract

Thrombosis is a major complication of cardiovascular disease, leading to myocardial infarction, acute ischemic stroke, or venous thromboembolism. Thrombosis occurs when a thrombus forms inside blood vessels disrupting blood flow. Developments in thrombectomy to remove thrombi from vessels have provided new opportunities to study thrombus composition which may help to understand mechanisms of disease and underpin improvements in treatments. We aimed to review thrombus compositions, roles of components in thrombus formation and stability, and methods to investigate thrombi. Also, we summarize studies on thrombus structure obtained from cardiovascular patients and animal models. Thrombi are composed of fibrin, red blood cells, platelets, leukocytes, and neutrophil extracellular traps. These components have been analyzed by several techniques, including scanning electron microscopy, laser scanning confocal microscopy, histochemistry, and immunohistochemistry; however, each technique has advantages and limitations. Thrombi are heterogenous in composition, but overall, thrombi obtained from myocardial infarction are composed of mainly fibrin and other components, including platelets, red blood cells, leukocytes, and cholesterol crystals. Thrombi from patients with acute ischemic stroke are characterized by red blood cell- and platelet-rich regions. Thrombi from patients with venous thromboembolism contain mainly red blood cells and fibrin with some platelets and leukocytes. Thrombus composition from patients with myocardial infarction is influenced by ischemic time. Animal thrombosis models are crucial to gain further mechanistic information about thrombosis and thrombus structure, with thrombi being similar in composition compared with those from patients. Further studies on thrombus composition and function are key to improve treatment and clinical outcome of thrombosis.

**Highlights**

- Thrombosis occurs when a thrombus forms inside blood vessels disrupting blood flow.
- Thrombi obtained from myocardial infarction are composed of mainly fibrin and other components, including platelets, red blood cells, leukocytes, and cholesterol crystals.
- Thrombi from patients with acute ischemic stroke are characterized by red blood cell- and platelet-rich regions.
- Thrombi from patients with venous thromboembolism contain mainly red blood cells and fibrin with some platelets and leukocytes.
- Thrombus structure and composition are important for risk of thrombosis and thrombus removal.

Changes in clot structure are of key interest due to associations with risk of myocardial infarction (MI), acute ischemic stroke (AIS), and venous thromboembolism (VTE). Most studies explored links between in vitro clot structure and thrombosis.^1^ Recent literature exceeds 1000 publications, with >400 in the past 5 years alone.^1–3^ However, studies into the structure and components of thrombi formed in vivo remain limited. With the advent of new methodologies and imaging techniques, in vivo or ex vivo thrombi obtained by thrombectomy can be studied in much greater detail than ever before. Recent studies have been taking this approach to shed light on how in vivo thrombus structures relate to thrombosis in different vascular beds. In this review, we will summarize the main findings from these studies, their technological aspects, associations with disease, insights from animal models, and highlight key areas for future research.

## Major Thrombus Components

Formation of thrombi leads to vessel occlusion or the generation of emboli that block blood vessels further downstream, resulting in MI, AIS, or pulmonary embolism (PE). Principal components of thrombi include fibrin, platelets, red blood cells (RBCs), leukocytes, and neutrophil extracellular traps (NETs). However, the relative contribution of each component differs between thrombus location and disease pathology. Below we discuss each component and how they contribute, followed by differences in thrombus composition between different thrombotic diseases.

### Fibrin(ogen)

Fibrinogen is a 340 kD glycoprotein that circulates in blood at 2 to 5 mg/mL.^2^ When coagulation is triggered, thrombin cleaves fibrinogen into fibrin that polymerizes into a network of fibers,^3^ stabilizing blood clots. Fibrin is a major contributor to thrombi, with changes in its structure known to affect clot formation, stability, and breakdown. High thrombin concentrations lead to dense fibrin networks that are relatively resistant to fibrinolysis.^2^ Previous in vitro studies have linked changes in fibrin clot structure,^1^ viscoelastic properties,^4^ and hypofibrinolysis^5^ to thrombosis. However, despite the consistent link between in vitro clot structure and thrombosis, it is still unclear whether comparable changes are reflected in the structure of in vivo thrombi. Early studies that explored in vivo thrombi used angioscopy to evaluate macroscopic properties. Two main types of thrombi were observed in patients with acute coronary syndromes, white and red.^6^ Histology indicated that white thrombi from patients with ST-segment–elevation MI (STEMI) were mainly composed of fibrin, whereas red thrombi were mainly composed of RBCs.^7^ Thrombi from patients with STEMI that are resistant to fibrinolysis are characterized by dense fibrin and higher contents of platelets and VWF (von Willebrand factor).^8^ These studies highlight how fibrin contribution to thrombi varies between disorders and may impact disease progression and outcome. In vitro studies have shown how clots with increased fibrin demonstrate greater friction,^9^ suggesting these thrombi are stickier. This could be an important factor in clot stability, embolization, and thrombectomy.

**Figure 1.**
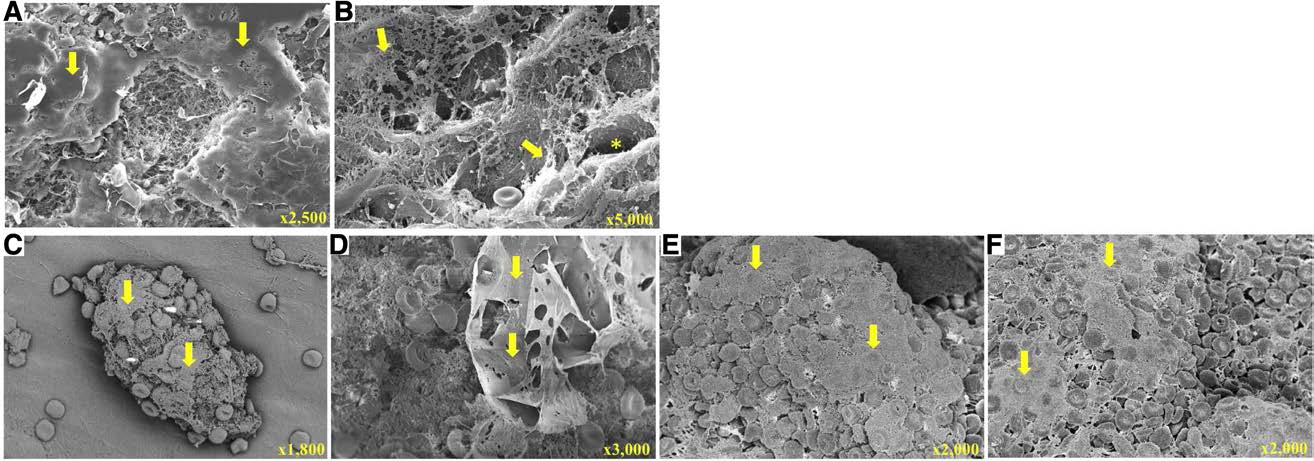
**Fibrin biofilm in thrombi.A** and **B**, Film structures in thrombi extracted from patients with abdominal aortic aneurysm. **A**, Fibrin film covering the thrombus (arrows). **B**, Film transitioning from film to fibers (arrows) and film lining channels traversing the thrombus (asterisk). **C–F**, Thrombi from murine thrombosis model (FeCl_3_ injury of the inferior vena cava) after perfusion/fixation. **C**, Small thrombus showing partial film coverage (arrows). **D**, Film covering part of the thrombus (arrows). **E** and **F**, Film coverage localized on top of red blood cells (RBCs; arrows).

Recent findings presented a new structural feature of fibrin in clots. Instead of forming 3-dimensional fiber networks, fibrin molecules align into continuous films forming a protective layer across the surface of clots, providing protection against infection.^10^ There is evidence that fibrin also forms films within the vasculature. Images of intraluminal thrombi from patients with abdominal aortic aneurysm show signs of fibrin film both within and on the clot surface (Figure 1). Clots from murine venous thrombosis models also demonstrate the presence of film (Figure 1). In agreement with these unpublished findings, many studies have presented evidence of film in thrombi. Scanning electron microscopy (SEM) micrographs of thrombi from patients with PE,^11^ AIS,^12^ or MI^13^ showed fibrin film in these thrombi. A recent study showed structures surrounding thrombi removed from patients with AIS that had similar properties to fibrin films, which these authors called a shell, and which slowed thrombolysis.^14^ Combined with the findings that fibrin content varies in thrombi, these data indicate an important role for fibrin in thrombus characteristics, influencing stability, embolization, and breakdown. Further research is needed to understand how fibrin content in thrombi influences disease onset, progression, and outcome, and how previously described changes in in vitro fibrin clot structure relate to in vivo thrombus structure.

### Platelets

Platelets are an attractive target for antithrombotic treatment. Activated platelets provide negatively charged membrane surfaces that are essential for assembly of the prothrombinase and tenase complexes.^15^ Platelets form different populations during clot formation, including procoagulant platelets which support thrombin generation and fibrin formation, and aggregating platelets involved in initial clot formation and contraction.^16^ Within thrombi, strongly activated platelets localize in the inner core, while discoid and quiescent platelets localize to the exterior.^16^ Clot contraction is mediated by aggregating platelets binding fibrin fibers via α_IIb_β_3_.^16^ Procoagulant platelets show a balloon-like structure, exposing phosphatidylserine on the surface to generate thrombin in situ.^17^ In addition, platelets promote thrombus growth and propagation through glycoprotein VI binding to fibrin.^18,19^ More studies are needed to investigate the temporal and morphological contribution of platelets to the architecture of thrombi obtained ex vivo.

### Leukocytes

Leukocytes contribute significantly to clot formation and are found in both arterial and venous thrombi.^20,21^ Leukocytes bind fibrin via integrin receptor α_M_β_2_ (Mac-1), which supports the inflammatory response.^22^ Neutrophils, the most abundant leukocyte in circulation, release matrix metalloproteinases, platelet-activating factor, cathepsin G, and elastase.^23,24^ These molecules can impact coagulation via a number of mechanisms, including activation of coagulation factor V, factor VIII, and factor X,^25–27^ activation and aggregation of platelets,^28^ degradation of antithrombin III and proteolytic cleavage of TF (tissue factor) pathway inhibitor.^29,30^ Monocytes are a major source of intravascular TF expression and provide a membrane surface for coagulation initiation in a number of conditions.^25,31^ TF is also expressed by neutrophils in animal models.^32^ Some studies report TF expression in neutrophils and eosinophils,^33,34^ but other studies fail to detect TF expression in granulocytes.^35,36^ Some of these discrepancies may be attributed to direct transfer of TF from monocytes to granulocytes.^37^

### Neutrophil Extracellular Traps

A key mechanism by which neutrophils contribute to thrombus composition involves generation of NETs. NETs are formed by neutrophils extruding DNA, histones, and granular proteins in response to microbial invasion, inflammatory stimuli, or activated platelets.^38^ Increasing evidence shows that NETs are associated with thrombosis.^39,40^ NETs are thought to trigger coagulation via the intrinsic pathway, with DNA acting as scaffold for contact assembly triggering thrombin generation.^38^ Heparin binds histones in NETs resulting in their breakdown and reduction of thrombosis.^41,42^ One study suggested that neutrophil DNA or histones can trigger coagulation, but not NETs, due to histone-histone and histone-DNA interactions.^43^ NETs act as scaffold for platelet aggregation, further promoting thrombus development, and there is evidence they increase resistance to thrombolysis.^38,41,42^ The relationship between NETs and thrombosis and the underlying mechanisms remain of key interest since NETs are present in both arterial and venous thrombi.^39^ Extracellular DNA has been observed in platelet-rich areas of AIS thrombi but not in RBC-rich regions.^44^ However, NET-like structures were found in RBC-rich regions of murine venous thrombi,^39^ highlighting the need for further research into the role and localization of NETs.

### Red Blood Cells

Recent studies have indicated that RBCs play a more functional role in clot structure and function than previously thought. FasL/FasR (CD95) receptor ligand interactions between RBCs and activating platelets have been shown to lead to phosphatidylserine exposure on both platelets and RBCs and to platelet degranulation, contributing to thrombus formation.^45,46^ RBCs have been shown to support thrombin generation via the meizothrombin pathway.^47^ High hematocrit promotes accumulation of platelets at vascular injury sites by pushing platelets from the blood vessel center to the vessel wall.^48^ RBCs retention within venous thrombi is mediated by factor XIII, suggesting that targeting factor XIII to reduce RBCs contents could be a therapeutic approach in venous thrombus as less RBC content may limit thrombus mass and stability.^49^ RBCs are normally biconcave, however, recent studies show that RBCs adopt an alternative structure during thrombosis called polyhedrocytes.^50^ Forces generated by platelets pulling on fibrin fibers lead to clot contraction, compressing RBCs together, forcing them into a polyhedral structure.^50^ Polyhedrocytes have been detected in thrombi from patients with STEMI.^50^ Other studies indicate that polyhedrocytes are present in 20% to 31% of thrombi from patients with MI.^51,52^ Polyhedrocytes are also found in venous thrombi, pulmonary emboli, and cerebral thrombi.^53,54^ RBCs in clots affect fibrinolysis^55^ and alter clot mechanical properties.^56^ Furthermore, disorders such as sickle cell disease make RBCs rigid,^57^ reducing thrombus permeability.^58^ RBC-rich thrombi contain more inflammatory cells than other thrombi and associate with increased thrombus burden and impaired reperfusion in patients with STEMI.^59^ Altogether, 2 key mechanisms by which RBCs influence thrombosis are the formation of polyhedrocytes and the generation of additional thrombin. Better understanding of the mechanisms underpinning polyhedrocyte formation may lead to new treatments of thrombosis.

### Other Components

VWF, produced by megakaryocytes and endothelial cells, stabilizes factor VIII and mediates platelet adhesion, thereby supporting thrombosis.^60^ VWF has been detected in ex vivo thrombi from patients with MI and AIS.^14,61^ While the role of VWF in thrombosis through platelet activation and thrombin generation is well characterized, its presence and role(s) in thrombi require further investigation. The fibrinolysis pathway also plays a central role in thrombosis. tPA (Tissue-type plasminogen activator) converts plasminogen to plasmin, a primary fibrinolytic protease.^1^ tPA and its inhibitor, PAI-1 (plasminogen activator inhibitor-1), have been detected in thrombi from patients with MI.^8^ PAI-1 and protease nexin-1 have also been detected in AIS thrombi.^14^ Variation in fibrinolytic proteins and their inhibitors incorporated in thrombi may impact on resistance to therapeutic thrombolysis. Finally, cholesterol crystals are present in thrombi obtained from patients with MI which were mainly derived from plaque rupture.^62^ The role of cholesterol crystals in thrombi is unknown and requires further study.

## Methodologies to Investigate Thrombus Structure

Techniques to investigate thrombus structure have their advantages and limitations (summarized in the Table). SEM has been used in several studies.^52,62,63^ It offers high-resolution images and visualization of major thrombus components, providing descriptive and semiquantitative data.^52,62^ This enables identification of thrombus components, such as polyhedrocytes^50^ and fibrin fibers or films.^10^ SEM supports the analysis of platelet morphological alterations, such as aggregation and pseudopod formation.^70^ In addition, SEM is very useful for the analysis of fibrin properties within a thrombus, such as fibrin coverage area and fibrin fiber diameter. However, NETs are structurally difficult to differentiate from fibrin using SEM,^71^ highlighting the need for combining SEM with other methods using specific labeling such as correlative light and electron microscopy, laser scanning confocal microscopy (LSCM), or immunohistochemistry to confirm the nature of some of the components analyzed.

**Table.**
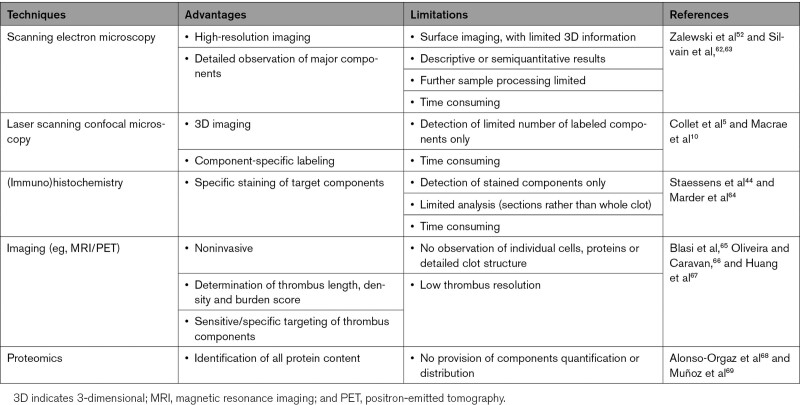
Methods Used to Investigate Thrombus Structure

Once thrombi are prepared for SEM no further analysis (eg, histology) can be performed.^62,63^ Identification of components is based on morphological appearance, without specific staining. Furthermore, limited 3-dimensional information is obtained by SEM. SEM may induce artifacts due to sample processing, fixation and dehydration. Nevertheless, SEM procedures have been refined to keep artifacts to a minimum, and in most cases, negligible. In contrast, LSCM provides 3-dimensional images in fully hydrated conditions.^5,10^ Fluorescent antibodies can be used to label specific proteins, allowing for identification of components. However, extracted thrombi need to undergo fixation and chemical clearing before LSCM imaging.^72^ Resolution is lower than SEM and only labeled components are detected with a finite number of fluorophores used at once. RBCs within a thrombus may hinder optical access to the inside of the clot and thus the collection of deep, high-resolution 3-dimensional images by LSCM.^72^ New optical clearing methods may need to be explored to produce a suitably transparent thrombus to allow for deep imaging of thrombi at the micrometer scale.^72^ This method could be effective for imaging the thrombus structure of patients. LSCM and SEM can each be used sequentially on the same sample, but both are expensive and time consuming. Correlative light SEM may provide future opportunities and new developments in the field through matching confocal and electron imaging of the same thrombus area.^73^

Immunohistochemistry has been used to identify specific components in thrombi,^44,64^ via sectional analysis of thrombi with a range of resolutions (nm-µm). Recent developments allow improved imaging of thrombi and their constituents.^39^ However, despite analysis of sections, mostly 2- rather than 3-dimensional information has so far been obtained. Furthermore, the preparation of thin sample slices may damage the sample to be analyzed. A combination of imaging techniques is recommended to compare high-resolution methods such as SEM with methods that allow specific staining, such as immunohistochemistry and LSCM. Noninvasive, sensitive, and specific imaging techniques, including magnetic resonance imaging (MRI) and positron-emitted tomography, have also been used to study thrombus composition in patients and animal models.^65,66^ Imaging of AIS thrombi revealed information about clot length, clot density, and clot burden score.^67^ Other studies used proteomic approaches to identify thrombi constituents.^68,69^ Correlating thrombus proteome to clinical features could be useful for AIS cause identification, which may help selecting appropriate treatment.^74^

## Thrombus Composition by Pathology

**Figure 2.**
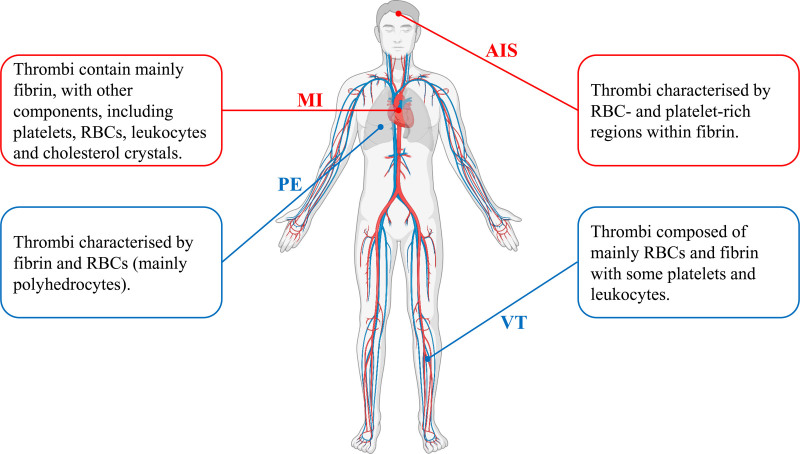
**Thrombus composition in myocardial infarction (MI), acute ischemic stroke (AIS), venous thrombosis (VT), and pulmonary embolism (PE).** RBC indicates red blood cell. Created with BioRender.com.

Since the development of thrombectomy and other endovascular approaches including balloon angioplasty, endovascular thrombolysis, and stenting, mortality of patients with thrombosis (particularly MI and AIS) has decreased substantially. With the advent of thrombectomy new opportunities have emerged to investigate pathological differences in thrombi extracted from patients. Several studies have assessed thrombus composition using a combination of SEM, LSCM, and immunohistochemistry (summarized in Figure 2).

### Venous Thromboembolism

VTE is caused by thrombi in the deep veins of the limbs, which can travel to the lungs causing PE. VTE is triggered by 3 fundamental mechanisms, endothelial dysfunction, altered blood flow, and hypercoagulability, also called Virchow’s Triad.^75^ Data on thrombi obtained by thrombectomy from patients with VTE is limited. A single case study showed that an embolus from a PE patient was composed of fibrin with RBCs and a small number of platelets.^11^ SEM indicated structures similar to fibrin film covering the embolus, although these were not commented on at the time. In another case-study of a patient with chronic venous insufficiency, thrombi were aspirated from the right atrium and pulmonary arteries. The atrial thrombus contained mostly RBCs, with platelets, a small number of leukocytes, and a random arrangement of fibrin fibers. In comparison, more fibrin and platelets aggregates were observed in pulmonary thrombi, with fibrin fibers arranged along the vessels.^76^ An earlier review showed a venous thrombus with densely packed RBCs resembling polyhedrocytes interspaced by fibrin.^77^ Furthermore, an autopsy study examined venous thrombi and pulmonary emboli from 8 patients who died from VTE, showing that all thrombi and emboli contained fibrin, RBCs, VWF, and α_IIb_β_3_.^78^

A study of the composition and mechanical properties of 2 emboli obtained from a patient with PE showed that their structure was heterogeneous, with one embolus containing more RBCs but less fibrin than the other.^79^ Cyclic compression analysis showed that the fibrin-rich embolus exhibited a higher stress response than the RBC-rich embolus. These findings indicate that thrombus composition impacts on mechanical properties, which may affect embolization, endovascular removal by thrombectomy and thrombolysis. A recent study compared thrombi from patients with STEMI (n=45) and venous thrombi (n=25) both obtained by open thrombectomy to pulmonary emboli from autopsies (n=10).^53^ All arterial thrombi were composed of fibrin followed by platelets, while major components of venous thrombi and pulmonary emboli were RBCs followed by fibrin. However, pulmonary emboli showed more fibrin and fewer RBCs than venous thrombi.^53^ In addition, RBCs in pulmonary emboli were present in the form of polyhedrocytes. The structure of fibrin in venous thrombi was heterogenous, including fibers, sponge, and bundles, while most of the fibrin in pulmonary emboli were fibers.^53^ The mechanisms behind these differences in composition are not known. It is possible that thrombus areas with a particular composition embolize, or emboli may change composition after they embolize and lodge in the pulmonary circulation. In view of the relative paucity of data, further studies investigating thrombus composition in VTE are required, however, thrombectomy is not normally a treatment of choice in VTE. Future developments in imaging and in vivo models are needed to progress this area of research.

Animal models of VTE show changes in thrombus composition and susceptibility to fibrinolysis over time.^80,81^ However, research on thrombus maturation in humans is limited. An autopsy study has used immunohistochemistry and LSCM to examine the thrombus composition in 140 cases of subjects who died due to PE.^82^ Upon autopsy, thrombi were classified into phase 1 (first week), phase 2 (second to eighth week) and phase 3 (older than 2 months). Phase 1 thrombi were composed of fibrin, platelets, agglomerated RBCs, and leukocytes. There was no interaction between the thrombus and the vascular endothelium. In phase 2, thrombi showed penetration of fibroblasts, and endothelial sprouting became apparent. Furthermore, macrophages containing predominantly hemosiderin, RBCs and fibrinous transformation were observed with nuclear debris of leukocytes. In phase 3, the thrombi became hyalinized, and few leukocytes were present interspersed by fiber-rich and cell-deficient connective tissue.^82^

A catherization study analyzed thrombi extracted from 17 patients with deep vein thrombosis and 10 patients with PE. Thrombi were classified into stage 1 (0–1 day old; composed of fibrin, platelets, RBCs, and neutrophils), stage 2 (1–3 days old; acute thrombi containing inflammatory cells without cellular organization), stage 3 (4–7 days old; thrombi exhibiting cellular growth, including smooth muscle cells and endothelial cells), and stage 4 (>7 days old; healing thrombi characterized by layers of smooth muscle cells, proteoglycan depositions, and endothelial filtration). All thrombi contained fibrin, RBCs, platelets, and inflammatory cells, and thrombi were generally younger in PE than patients with deep vein thrombosis.^83^ Based on the relatively scant literature on thrombus maturation in VTE, further clinical and preclinical research is necessary to gain clearer insights into how thrombi change over time.

### Myocardial Infarction

MI is caused by rupture of an atherosclerotic plaque resulting in thrombosis.^84^ Thrombi extracted from patients with STEMI (n=44) were analyzed by SEM and contained mainly fibrin (60%), with the remainder (40%) composed of platelets, RBCs, cholesterol crystals, and leukocytes.^62^ Another study of sudden cardiac death (n=23) and STEMI (n=98) showed similar results, with no difference observed between these 2 groups.^63^ A separate study also showed that the major component of STEMI thrombi (n=40) was fibrin (49.1%), with other components, including RBCs (24.2%), platelets (11.6%), and leukocytes (3.7%).^85^ Immunobiological analysis of MI thrombi showed the presence of monocytes, neutrophils, and lymphocytes.^86^ Acute MI thrombi (n=29) analyzed by immunohistochemistry showed that thrombi contained fibrin, platelets, RBCs, and leukocytes.^87^ Other immunohistochemistry analysis of occlusive MI thrombi (n=15) revealed the presence of fibrin, α_IIb_β_3_, TF, and VWF.^61^ Coronary arteries from patients with MI (n=31) examined postmortem showed that thrombi associated with ruptured plaques have more fibrin (74%) than platelets (35%), while thrombi associated with eroded plaques have more platelets (70%) than fibrin (51%). Tissue factor contributed more to thrombus formation in plaque rupture than erosion.^88^ Antimicrobial peptides released by leukocytes have been shown to contribute to platelet activation and thromboinflammation in human and murine models of MI.^89^

Thrombi surfaces contain more fibrin and platelets, and fewer RBCs, than their inner parts.^52^ Inner parts of STEMI thrombi are rich in polyhedrocytes,^50–52^ providing a densely packed structure resistant to fibrinolysis. A comparison between thrombi from patients with STEMI and peripheral arterial disease indicated reduced fibrin content, fibrin fiber diameter, and fibrin/platelet ratios in the coronary thrombi.^90^ A retrospective study exploring fibrin films in thrombi from patients with STEMI showed that fibrin film was detected on ≈15% of thrombi.^13^ Film was not detected in all thrombi due to heterogeneity of thrombi and could also have been missed due to the study being retrospective.

**Figure 3.**
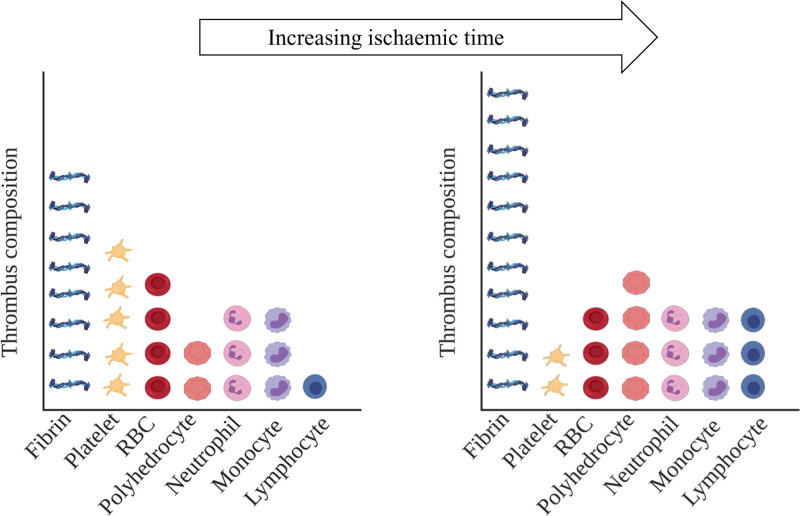
**Ischemic time and thrombus composition in patients with ST-segment–elevation myocardial infarction (STEMI).** The amounts of fibrin, polyhedrocytes, and lymphocytes increase with ischemic time, while platelet and red blood cell (RBC) loads decrease. Note: the ratios of components within each thrombus (individual graph), as well as the ratios of components between both thrombi (between graphs), are representative but not quantitative. Created with BioRender.com.

Ischemic time has been reported to affect the composition of thrombi (Figure 3). Silvain et al^62,63^ demonstrated that as ischemic time increased the amount of fibrin increased while platelets decreased in STEMI thrombi.^62,63^ STEMI thrombi (n=40) retrieved over 12 hours after the onset of symptoms showed more fibrin than thrombi retrieved within 3 hours.^85^ Correspondingly, RBCs decreased over time, but no associations were found for leukocyte and platelet counts.^85^ STEMI thrombi (n=65) retrieved >6 hours after onset of symptoms showed more compact fibrin network than thrombi retrieved <3 hours.^86^ With increasing time after symptom onset, platelet numbers decreased, and lymphocyte numbers increased.^86^ Polyhedrocyte formation increased with ischemic time in thrombi from patients with MI.^51^ Taken together, these findings indicate that thrombus composition may change over time after initial vessel occlusion in STEMI. Such changes in thrombus composition may have important implications for mechanical thrombectomy, thromboaspiration, and thrombolysis. Further research is required to understand the mechanisms through which thrombi change structure, for example, through thrombus component reorganization or clotting-lysis cycles.

### Acute Ischemic Stroke

AIS, the most common type of stroke, is caused by thrombosis in the cerebral circulation. AIS thrombi are due to atherosclerosis or cardiac embolism and result in disrupted blood flow in the brain and subsequent neurological disorder.^91^ A recent study showed that AIS thrombi (n=177) obtained by thrombectomy contain 2 distinct structural areas, platelet- and RBC-rich regions, that interspersed each other throughout the thrombi.^44^ Platelet-rich regions contained dense fibrin, platelets, VWF, leukocytes, and extracellular DNA. RBC-rich regions, however, were composed of RBCs and fibrin, while bordered by platelets and leukocytes.^44^ Another recent study showed that AIS thrombi (n=199) presented with a surface structure that resembles fibrin films.^14^ They referred to this as an outer shell, mainly composed of fibrin, that slowed thrombolysis. One complication in patients with AIS is thrombus migration, whereby the clot travels downstream in the cerebral vasculature, resulting in worse outcomes. Migrating thrombi contained more RBCs and less fibrin/platelets than stable thrombi.^91^ This may relate to previous findings that RBC-rich clots are less sticky.^9^ In addition, fibrin anchors clots to the site of vascular lesion, thus preventing embolism in animal models.^92^

Histological analyses showed that AIS emboli (n=25) were composed of platelets and fibrin with RBC-rich regions and leukocytes, including monocytes and neutrophils.^64^ A further histological study of AIS thrombi (n=37) showed that inflammatory T cells and monocytes were associated with RBC-rich clots, while VWF was associated with fibrin-rich clot.^93^ Early signs of vessel damage, including hyperdense middle cerebral artery sign and blooming artifact by computed tomography and MRI, indicate presence of thrombus.^94^ Thrombi from hyperdense middle cerebral artery sign and blooming artifact patients were RBC rich, while thrombi lacking these signs were fibrin rich.^94^ AIS thrombi (n=40) have been histologically categorized into early phase (RBC proportion dominant or equal to fibrin) and late phase (fibrin dominant and organized fibrin). Presence of hyperdense artery sign has been associated with early phase thrombus.^95^ Cerebral thrombi from patients with AIS (n=41) were composed of areas with many RBCs, mainly polyhedrocytes, and fibrin mixed with platelets with the presence of few leukocytes. There was no significant difference in RBC content between patients with cardioembolic and atherothrombogenic stroke. However, fibrin content was higher in patients with cardioembolic than patients with atherothrombogenic stroke.^54^ Similarly, previous studies showed that AIS of cardioembolic cause was associated with fibrin-rich thrombi while noncardioembolic thrombi (atherothrombogenic and cryptogenic thrombi) were associated with RBC-rich thrombi, however, the mechanisms behind these differences are unknown.^96^ Together, these studies indicate that thrombus composition changes by AIS cause, with RBC-rich thrombi associating with thrombus migration and fibrin-rich thrombi associating with stable and late phase thrombosis. However, one autopsy study found that the RBC content in cardioembolic thrombi was higher than in atherothrombotic stroke, indicating that the differences by cause may be more nuanced.^97^ The association of these types of thrombi with disease outcome and treatment such as thrombectomy and thrombolysis deserve further study, including detailed analysis of the composition of these thrombi in terms of other components using state-of-the-art methodologies.

### Therapeutic Implications

Thrombosis treatments include the use of antiplatelet, anticoagulant, and fibrinolytic agents. Antiplatelets are generally used to prevent and treat arterial thrombosis,^98^ which is caused by atherosclerotic plaque rupture that leads to collagen exposure followed by platelet aggregation and thrombus formation.^99^ Treatments targeting platelets appear effective in this setting as platelets have an important role in arterial thrombus growth.^99^ However, venous thrombosis which occurs under low shear stress and is largely driven by a coagulation imbalance is treated with anticoagulants since venous thrombi contain an abundance of fibrin.^98,99^ Fibrinolytic agents may be used in both arterial and venous thrombosis settings.^98^

Many trials have indicated that a combination of anticoagulant and antiplatelet treatments could be an effective strategy for mitigating cardiovascular disease.^100^ In one study, patients with acute coronary syndrome were treated with either a combination of antiplatelets agents and rivaroxaban (an activated factor X inhibitor) or a placebo. The use of rivaroxaban reduced instances of cardiovascular death, MI, and AIS; however, it also increased the risk of major bleeding, including intracranial hemorrhage.^101^ In another study, coronary syndrome patients were treated with either aspirin, a combination of rivaroxaban and aspirin, or rivaroxaban without aspirin. Among these groups, the combination of rivaroxaban and aspirin yielded the best cardiovascular outcomes but was associated with a moderate increase in bleeding risk.^102^

Therapeutic fibrinolysis is centered around plasminogen into plasmin conversion in the thrombus using tPA or tPA analogues.^103^ Increasing evidence indicates that thrombolysis may be facilitated by targeting additional components in the thrombus other than fibrin. For instance, NETs that are present in venous and arterial thrombi and have been shown to delay thrombolysis by tPA could be a potential therapeutic target using DNases.^104,105^ Furthermore, the effectiveness of thrombectomy could be influenced by thrombus compositions. For example, in patients with AIS, it has been shown that RBC-rich thrombi are associated with successful recanalization,^106,107^ require fewer passes and lower procedure time than fibrin-rich thrombi.^96^ Thus, analysis of the thrombus structure in cardiovascular patients could aid the development of new thrombolytic or antithrombotic strategies.

## Insights From Animal Models

Animals models are essential for the study of thrombosis pathophysiology or the role of drugs in thrombosis prevention.^108–110^ Studies discussed below provide insight in the composition of arterial or venous thrombi.

### Animal Models of Arterial Thrombosis

An early study by Randall and Wilding^111^ showed that thrombi from rabbits induced by electrical stimulation of the wall of carotid arteries were platelet rich.^111^ Clots formed in dog femoral arteries showed that tPA-induced thrombolysis of platelet-rich thrombi induced by transluminal electrode was impaired compared with that of fibrin-rich thrombi induced by intraluminal copper wire and that reocclusion under antiplatelet therapy was more frequent for fibrin-rich thrombi.^112^ Complementary to this, studies in rabbit femoral arteries showed that RBC-rich thrombi triggered by thrombin infusion were more prone to thrombolysis than platelet-rich thrombi induced by everted artery graft, with platelets providing a source of PAI-1.^113^ A later study in rat carotids showed that platelet-rich thrombi (formed by photochemical injury) were more prone to embolization causing cerebral infarcts, than fibrin-rich thrombi (formed by balloon catheter denudation).^114^ These studies indicate an important role for thrombus composition in the severity of thromboembolic diseases. FeCl_3_ injury of rat carotid arteries showed initial platelet clumping on the denuded endothelium, while occlusive thrombi consisted of RBCs and leukocytes tightly packed by a fibrin mesh, with tightly adhered platelets at the anterior side, highlighting heterogeneity of arterial thrombi.^115^ A pig carotid artery thrombosis model induced by balloon angioplasty showed that thrombi were heterogenous in the first 24 hours, including fibrin-rich and platelet-rich areas, and sporadic RBCs and neutrophils. Fibrin-rich areas were found at the thrombus and vessel wall interface. At day 1, thrombi consisted of granulated platelets, cellular debris, and compacted fibrin, while at 2 weeks, connective tissue was detected alongside cellular debris and unresolved fibrin. Over 3 to 9 weeks, thrombi became more fibrous, containing new blood vessels.^116^ This model may be relevant to human pathophysiology, particularly about thrombus composition and changes in chronic coronary occlusion.^116,117^

Occlusive thrombi from rabbit arteries by balloon angioplasty stained positive for α_IIb_β_3_, fibrin, VWF, and TF,^118^ in agreement with human studies.^119^ The neointima could play a role in thrombus composition and size due to tissue factor expression on the smooth muscle cell and macrophage-rich neointima, while small, platelet-rich thrombi formed on normal intima.^120^ A recent study indicated that mouse carotid artery thrombi triggered by FeCl_3_ were similar in composition when compared with human coronary thrombi, as both were heterogeneous with compact cell-rich regions, and less dense areas with fewer cells. NETs were present in both human and murine thrombi, with similar NETs to leukocyte ratio. Inhibition of NETs resulted in decreased thrombosis and reduced infarct size.^121^ Staining was targeted at particular components, and RBCs were partly overlooked in this study.

Human arterial thrombi mainly result from atherosclerosis and subsequent atherosclerotic plaque rupture, which rapidly generates a clot leading to MI and AIS. However, in general, most animal models of atherosclerosis do not develop thrombosis due to plaque rupture.^122^ Triggering thrombus formation, for example, by FeCl_3_ injury only replicates the final stages of atherothrombotic disease.^123^ Nevertheless, FeCl_3_ or needles have been used to induce thrombus formation in atherosclerosis-relevant models (eg, mice deficient in apolipoprotein E).^124–126^ Analysis of the corresponding thrombi compositions in relevant models for atherosclerosis may provide new insights in thrombus structure in the context of cardiovascular disease. Future studies should focus on the role of RBCs in addition to other clot components, as well as address questions regarding thrombus heterogeneity, composition, and their role in outcomes and treatment.

### Animal Models of Venous Thrombosis

McGuinness et al^127^ showed that 1-day old thrombi generated after stenosis of rat inferior vena cava consisted of platelets, RBCs, leukocytes, and fibrin, with neutrophils being the main leukocyte. Monocytes located initially to the thrombus edge but were more evenly distributed in mature thrombi.^127^ RBC hyperaggregability induced by pluronic F98 treated RBCs correlated with thrombosis occurrence in a rabbit venous thrombosis model.^128^ Venous thrombi from baboon iliac veins induced by temporary balloon occlusion contained NETs, with diffuse staining of histones and extracellular DNA colocalizing with VWF.^42^ Murine inferior vena cava thrombosis models showed that NETs are primarily located in RBC-rich regions, and colocalized with VWF.^39^ In agreement with NETosis in murine venous thrombi, NETs are also present in human venous thrombi obtained by thrombectomy.^129^

One-day old murine venous thrombi generated by combined reduced flow and mechanical endothelial injury were RBC-rich in the center with fibrin deposition at the periphery as demonstrated by in vivo magnetization transfer and diffusion-weighted MRI coupled with Martius scarlet blue staining. After 1 week, the central part contained RBCs encapsulated in fibrin, while after 4 weeks thrombi were mainly collagen rich.^130^ This suggests that magnetization transfer and diffusion-weighted MRI are promising for determining thrombus age via its composition. Young thrombi were rich in fibrin and RBCs, while collagen fibers were present after 1 week after FeCl_3_ injury of rat carotid arteries and femoral veins as demonstrated by histology.^65^ Fibrin peaked after 1 day in both venous and arterial thrombi, and venous thrombi showed more fibrin than arterial thrombi after a week, which was also detected by positron-emitted tomography imaging using the fibrin-specific ^64^Cu-FBP8 probe.^65^ Imaging techniques could provide a useful clinical noninvasive tool for detecting thrombus and assessing thrombus age. More agents targeting thrombus components, mainly fibrin and platelets, have been validated in thrombosis models in vivo.^131–133^ For example, contrast agents (microparticles of iron oxide and antibody targeting activated α_IIb_β_3_) have been used to analyze platelets and to monitor thrombolysis by MRI in murine arterial thrombi induced by FeCl_3_.^131^ Contrast agent targeting fibrin (EP-2104R) allow MRI detection of fibrin content in thrombi and indicated thrombus susceptibility to thrombolysis in a murine venous thrombosis model induced by reducing blood flow and endothelial disruption.^132^ Another study used near-infrared fluorescence method with fibrin-targeted agent (FTP11-Cy7) in deep vein thrombosis models induced by FeCl_3_.^133^

Murine venous thrombosis models provide key data on the maturation of venous thrombus. Venous thrombi showed changes of thrombus components from 2 to 4 weeks, from fibrin-dominant to collagen-dominant thrombi with increasing infiltration of inflammatory and mesenchymal cells, and these changes were correlated with clot stiffness.^80^ Due to the decreasing fibrin content with age, fibrinolytic efficiency reduces with increasing thrombus age in murine venous thrombosis models.^81^

Furthermore, murine inferior vena cava stasis thrombi showed areas rich in RBCs, fibrin(ogen), and neutrophils, but also contained monocytes and macrophages, with leukocytes colocalizing with urokinase plasminogen activator. PAI-1 colocalized with platelets, while plasminogen and α_2_-antiplasmin were also present in venous thrombi.^134^ Taken together, these studies indicate that thrombus composition changes with age, which likely impacts on disease development and treatment.

### Choice of Thrombosis Model

Overall, animal models of arterial and venous thrombosis help gain valuable information on the content and structure of thrombi, with structural characteristics similar to human thrombi, thus offering opportunities for the development of new diagnostic and therapeutic tools. However, there is no single model representing all aspects of arterial and venous thrombosis, and at all stages of disease, while different methods of thrombosis induction can impact thrombus formation timing, composition and architecture. Unlike in patients, thrombus formation in animal thrombosis models mainly occurs in healthy vessels that are acutely injured.^109^ Developing new models that better reflect a diseased environment (eg, inflammation and metabolic disease) could further support improved characterization of thromboembolism and thrombus structure. Small models (eg, mice) showed their value for mechanistic insights into thrombosis, particularly in view of the relative ease of genetic modifications. However, future studies in larger animal models that are anatomically more similar (including the vasculature) to humans than smaller species may be of interest.^109^ Therefore, with each model offering its own benefits and limitations, it is important to carefully select thrombosis models and animal species based on study-specific objectives.

## Conclusions

The advent of thrombectomy to treat a growing number of diseases heralds a new era in thrombosis research. It has enabled the analysis of thrombi from patients in ever greater detail, thus learning new information about their individual make-up. Careful consideration should be given to methods of thrombus composition analysis as each has their advantages and disadvantages. Another important consideration is to complement component-specific staining-based techniques with other methods that provide structural information at high resolution so that no particular thrombus components may be overlooked. Based on the literature thus far thrombus composition clearly is heterogeneous, varying between thrombotic disorders and patients, but even within the same patient or thrombus. Areas for future research include the relationship between thrombus areas and arterial or venous thrombosis, and how thrombus composition changes over time. Other remaining questions include how thrombus composition associates with embolism, effectiveness of thrombectomy or thrombolysis, and the role of polyhedrocytes, fibrin films, or other new structures in thrombosis. The development of better techniques to investigate thrombus composition, including noninvasive imaging methods, and improved animal models that are more physiologically relevant to human disease, will further be beneficial for future improvements in prevention and treatment of this devastating disease.

## Sources of Funding

R.A.S. Ariëns is supported by grants from the BHF (RG/18/11/34036) and the Wellcome Trust (204951/B/16/Z). F.L. Macrae is supported by the Wellcome Trust (215861/Z/19/Z).

## Disclosures

None.
